# Expression level of matrix metalloproteinases -1, -2 and -9 associated with pelvic organ prolapse: experimental analytical study of round ligaments of Congolese women in two hospitals from the city of Kananga in Democratic Republic of Congo

**DOI:** 10.11604/pamj.2025.50.93.45722

**Published:** 2025-04-07

**Authors:** Antoine Tshimbundu Kayembe, Patrick Kahindo Muyayalo, Andy Mbangama Muela, Rahma Raschid Tozin

**Affiliations:** 1 Department of Gynaecology and Obstetrics, Faculty of Medicine, University Notre-Dame of Kasayi, Central Kasaï, Democratic Republic of Congo,; 2 Department of Gynaecology and Obstetrics, Faculty of Medicine, University of Kinshasa, Kinshasa, Democratic Republic of Congo,

**Keywords:** Matrix metalloproteinases-1, matrix metalloproteinases-2, matrix metalloproteinases-9, round ligaments, pelvic organ prolapse, Kananga

## Abstract

**Introduction:**

pelvic organ prolapse is a pathology of ligamentous connective tissues and matrix metalloproteinases are the main actors responsible for tissue remodeling by degrading connective tissue. The objective of this present study is to determine the expression rate of matrix metalloproteinases-1, -2 and -9 in prolapsed round ligaments and identify the types of matrix metalloproteinases most determining in the appearance of pelvic organs prolapse in in two pilot hospitals of Saint-Georges and of Bon-Berger of the city of Kananga in the Democratic Republic of Congo.

**Methods:**

we conducted an analytical experimental study based on immunohistochemical examination of round ligaments of 100 consenting patients divided in two groups with and without pelvic organ prolapse treated in the gynecology departments of Bon-Berger hospitals in Tshikaji and Saint-Georges in Katoka in city of Kananga, from January 1^st^ to July 31^st^, 2023. Non-probability convenience sampling helped us select cases. The ANOVA test, the Chi-square test, and the logistic regression with adjustment are used in the statistical analyses.

**Results:**

the average age of our patients with pelvic organ prolapse was 57.18 (SD: 8.17) years, and their average parity was 7.76 (SD: 1.04) delivery. This average parity was significantly increased compared to those of patients without prolapse. The expression rate of matrix metalloproteinases-1, -2, and -9 is significantly high in prolapsed round ligaments with a respective mean immunoreactive of 44.10± 22.04% (P=0.001), 39.46± 24.10% (P=0.001), and 39.34± 20.89% (P=0.001). Positive immunoreactivities to matrix metalloproteinases-1, -2, and -9 are significantly noted in patients with prolapse. There was a significant association between positive immunoreactivities to matrix metalloproteinase-1 (AOR 5.40, 95% CI: 0.981-29.794, P: 0.044) and matrix metalloproteinase-9 (AOR: 6.205, 95% CI: 1.467-26.239, P: 0.013) with the appearance of pelvic organs prolapses in the city of Kananga.

**Conclusion:**

the expression level of matrix metalloproteinases is increased in patients with pelvic organ prolapse and is associated with this condition, matrix metalloproteinases-1 and -9 are associated with the occurrence of pelvic organ prolapse. These results will serve as a basis for experimental research studies of inhibitory evidence of estrogens and progesterone on these matrix metalloproteinases in our city with a view to adding these hormones as a means of preventing pelvic organs prolapse in women at risk in our city of Kananga.

## Introduction

Pelvic floor disorders in women are mainly characterized by pelvic organ prolapse (POP), urinary or fecal incontinence, and sexual dysfunction and severely impact the quality of life of patients [1]. Pelvic organ prolapse is increasingly common due to population growth and the increase in the number of elderly people in the general population [2,3]. Demographic analyses have allowed Wu *et al*. [4] to predict that 9 million American women will be affected by POP in the USA by 2050. Prevalence of POP in the world varies from 2.90 to 97.70% depending on study approaches used. If the approach used is a symptom questionnaire, this prevalence is from 2.90 to 11.40% [5-14], but if we perform a clinical examination with POP quantification, it is from 31.80 to 97.70% [15-23]. The reported prevalence of POP worldwide is approximately 9.00%, and it is estimated to be closer to 20.00% in lower-income countries [24]. Several scientific works conducted in sub-Saharan African countries, such as Ghana [25], Gambia [26], Tanzania [27] and Ethiopia [28] have reported POP prevalences varying between 12.00 and 65.00%. In DR Congo, the prevalence of POP is set at 24.12% by a study that we conducted in the city of Kananga [29]. Pelvic organ prolapse is characterized by decreased collagen content, secondary to its massive degradation by Matrix metalloproteinases (MMPs), the level of which is significantly increased in pelvic tissues [30-36]. This leads to decreased tensile strength and weakening of pelvic tissues [37-39].

Matrix metalloproteinases are regulated by their tissue inhibitors (TIMPS) [33,34,40,41], cytokines of the inflammation such as interleukins [41], tumor necrosis factor-α (TNF-α) [42]; growth factors such as platelet derived growth factor [41], transforming growth factor β [42-46]; and inhibitory hormones such as: estrogens, progesterone [40,41,47] and corticosteroids [40,41]. According to a study conducted in our city of Kananga, factors associated with POP are intense physical work, fetal macrosomic, vaginal delivery, multiparty, obstetric trauma, menopause and malnutrition (BMI less than 18.5Kg/m^2^) [48] while in Kinshasa, multiparity, obesity, number of vaginal deliveries greater than 4, fetal macrosomia, obstetric trauma, menopause and vaginal delivery were factors associated with POP [49]. Several studies demonstrate that these factors would lead to POP by increasing high levels of MMPs to degrade collagen at pelvic level [35,46,50,51]. The lake of data concerning MMPs associated with POP in our city of Kananga justifies this present study. The objective of this study is to determine the expression level of matrix metalloproteinases (MMP)-1, -2 and -9 in prolapsed round ligament and identify types of MMPs associated with POP in two hospitals of Bon-Berger and Saint-Georges of city of Kananga in DR Congo.

## Methods

**Study design and setting:** this is an analytical experimental study comparing the study group composed of round ligaments in patients with POP to the comparative group composed of round ligaments in patients without prolapse (i.e. patients who suffer from other benign gynecological diseases), all having undergone total hysterectomy recorded during the mass campaign which was organized in the gynecological departments of two hospitals in the city of Kananga: Bon-Berger Hospitals and Saint Georges, from January 1^st^ to July 31^st^, 2023. These two hospitals are chosen because of the presence of trained and experienced medical staff, the high attendance of patients who suffer from POP, the more or less free management of POP through the various surgical care campaigns in the fistula cure account. Therefore, these 2 hospitals are references for the management of POP in our city of Kananga in DR Congo.

**Study population:** the population of our study consists of patients who have signed the informed consent form, aged between 40 and 79 years, who suffer from POP for the study group and other benign gynecological pathologies for the comparison group and who are treated during the mass campaign in the gynecology departments of Bon-Berger hospitals and Saint Georges in city of Kananga from January 1^st^ to July 31^st^, 2023, and matched according to age plus or minus 3 years. We used the non-probabilistic sampling of convenience for case selection. The limitation of our study in time and space determines the sample size. We included the different patients in this study according to the following criteria: patients who signed the informed consent form, aged between 40 and 79 years suffering from POP (study group) and other benign gynecological pathologies (comparison group) and who underwent total hysterectomy during the mass campaign in the gynecology departments of the Bon-Berger hospitals and Saint Georges in city of Kananga from January 1^st^ to July 31^st^, 2023. We excluded all patients who refused to sign the informed consent form, those who had already undergone surgery for POP, those suffering from malignant gynecological pathologies and those on hormone replacement therapy in case of complicated menopause. Our sample size was calculated using the following formula [48,50-52]


$$n\geq \frac{\left(1+\frac{1}{c}\right){\left(Z_{\alpha }+Z_{1}-\beta \right)}^{2}P\left(1-P\right)}{\left(P_{0}-p_{1}\right)},P_{1}=\frac{P_{0}\times OR}{1+P_{0}\left(OR-1\right)},P=\frac{P_{1}+CP_{0}}{1+c}$$

where: n= number of cases; c= number of subjects in the comparison group matched per study case; p0= expected proportion of exposed cases in the comparison group (0.0116); p1 = expected proportion of exposed cases in the study group (0.105); p= proportion of exposed cases in both groups (comparison and study) (0.058); Zβ = Z value for the first type risk (1.645); α = the risk of type I error (0.05); Z1-β = Z value corresponding to a surface equal to the power of the test (1 - β). The latter constitutes the probability of finding a significant difference (1.282) (1-β)= the desired power (0.9); OR= minimum OR that is set for the study to be of public health interest and estimated at 20 on the basis of studies on the Visco and Yuan model [52]. The calculated sample size is greater than 47 cases and we increased it to 50 cases for the study group and 50 cases for the comparison group. The number of cases in the comparison group is the product: cxn. A case compared is matched to a study case (c=1).

**Data collection:** we collected the data from patient interviews and searching patient medical records and gynecology department registers of two hospitals, and recorded in the data collection record. We used the following variables of study: age, parity, diagnosis, types of tissues sampled, MMP-1 expression level in grade, MMP-2 expression level in grade, and MMP-9 expression level in grade. Our data were collected as follows: after each patient admitted to this study has signed the informed consent form, information collection was done by interviewing the patients and searching medical records. A 10mm round ligament tissue biopsy was taken from each woman during surgery. The biopsy was fixed in 10% formalin and stored until immunohistochemical analysis.

**Immunohistochemical staining:** all our tissues collected were systematically fixed in 10% buffered formalin and transformed into paraffin blocks by routine methods. The sections of the samples at 5 µm thickness were made using the microtome, mounted on coated slides and stained using the double immunolabeling technique whose protocol is established in the anatomopathological laboratory of the University Clinics of Kinshasa. For each sample, we did hematoxylin and eosin staining (H&E) before immunohistochemical staining which used antibodies against MMP-1, MMP-2 and MMP-9.

**The immunohistochemical staining procedure:** after fixing the samples in 10% formalin and paraffin, these samples were sectioned at 5 µm thickness using a microtome. The paraffin sections mounted on the microscope slide were first incubated overnight at 60°C, then deparaffinized and finally hydrated in descending alcohol series; they were incubated in citrate at 96-97°C for 60 minutes, peroxidase activity was blocked by incubating the slide sections in 3% hydrogen peroxide (3% H_2_O_2_, 25 ml Universal DAB inhibitor, SN: 1950274, Ventana laboratories, Arizona, USA) for 15 minutes, we then incubated the slide sections in CuSO_4_ (Concentration: 5 g/l, view universal DAB Copper, SN: 2002356, Ventana laboratories, Arizona, USA) for 15 minutes, the sections were incubated in protein block for 15 minutes (3% bovine serum albumin in phosphate buffered saline =PBS) to block nonspecific binding or minimize nonspecific reactivity, we incubated the sections overnight at 4°C in a humidified chamber with the primary monoclonal antibodies against Human MMPs due to 3 slides per sample including one slide for MMP-1 antibody (concentration: 500 µg/ml, 25% agarose, anti-human MMP-1 monoclonal antibody, Sc-21731AC, Santa Cruz Biotechnology laboratories, Dallas, USA, dilution: 1: 100), one for MMP-2 antibody (concentration: 500 µg/ml, 25% agarose, anti-human MMP-2 monoclonal antibody, Sc-13595AC, Santa Cruz Biotechnology laboratories, Dallas, USA, dilution 1: 100) and one for MMP-9 antibody (concentration: 500 g/ml, anti-human MMP-9 monoclonal antibody, SC-12759AC, Santa Cruz Biotechnology laboratories, Dallas, USA, dilution 1: 100). These monoclonal antibodies were diluted in Dako Antibody Diluent (Glostrup, Denmark). We incubated these slides in the primary antibody amplifier mixed with hydrogen peroxide (concentration: 0.04% H_2_O_2_, 25 ml, Universal diaminobenzidine H_2_O_2_, SN: 1895381, Ventana laboratories, Arizona, USA) for 20 minutes, we proceeded to the incubation of the slides with the secondary antibodies (Universal HRP Multimer, 55 microgram of non-specific mouse immunoglobulin G per ml, SN: 1939485, Ventana laboratories, Arizona, USA) for 1 hour.

The slides were carefully washed with distilled water and then phosphate-buffered saline (PBS) after each step of the procedure. Finally, we incubated the slides with the chromogen which is a substrate of diaminobenzidine peroxidase (Universal DAB Chromogen 2%, SN: 20160923, Ventana laboratories, Arizona, USA) for 30 minutes, the sections were counterstained with hematoxylin for 5 minutes at room temperature, followed by rinsing with water and incubating in lithium carbonate to blue the hematoxylin. The sections were covered with Canada balsam and coverslip with bubble removal and drying for 1 hour, and mounted on the microscope (Olympus CX31) for examination at x200-400 magnification. To control the secondary antibody, sections were produced by omitting the primary antibody and showed no staining while the positive control slides were stained with non-specific mouse immunoglobulin G and there was no background reaction. Positive control: positive control slides for MMP-1, -2, and -9 were prepared with placental tissue. The monoclonal antibody was used similarly in the positive control tissue. Negative control: the negative control slide was prepared from the same tissue block as the sample. Being negative control, we performed the same staining method omitting the primary antibodies (which are replaced by phosphate-buffered saline).

**Figure 1:**
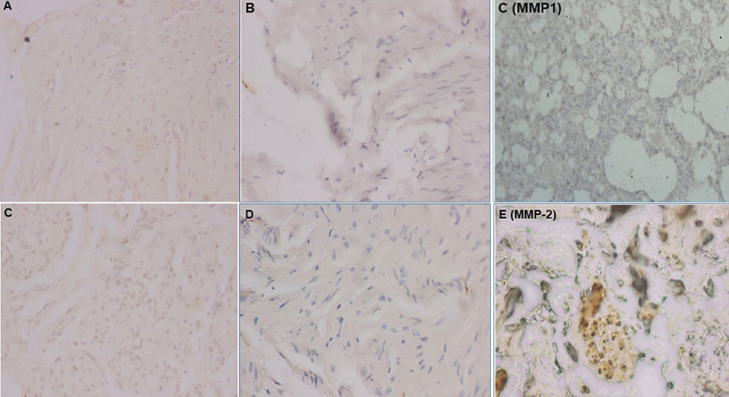
matrix metalloproteinases -1 and -2 immunoreactivity images: A) weak or focal matrix metalloproteinases -1 immunoreactivity (grade 1); B) moderate to diffuse matrix metalloproteinases -1 immunoreactivity (grade 3); C) diffuse matrix metalloproteinases -1 immunoreactivity (grade 4); D) local matrix metalloproteinases -2 immunoreactivity (grade 1); E) to moderate matrix metalloproteinases -2 immunoreactivity (grade 2) and; F) diffuse matrix metalloproteinases -2 immunoreactivity (grade 4)

**Figure 2:**
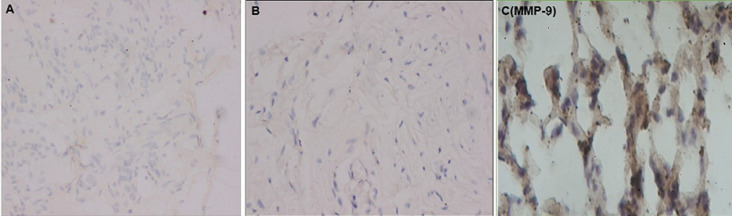
matrix metalloproteinases-9 immunoreactivity image: A) weak or focal immunoreactivity; B) focal to moderate immunoreactivity and; C) diffuse immunoreactivity

**Immunohistochemical analysis:** all our different slides were analyzed by an experienced pathologist to whom we concealed the information concerning the origin of the patient groups and of the samples (about a study or comparison patient). The recording of fibroblasts immunoreactive to MMP-1, -2, and -9 was done as a percentage. In addition, the percentages of immunoreactivity of the extracellular stroma were grouped in grades ranging from 0 to 4 (Figure 1, Figure 2).

**Operational definitions:** i) grade 0 corresponds to negative immunoreactivity; ii) grade 1 corresponds to 1 to 25% or focal immunoreactivity; iii) grade 2 corresponds to 26 to 50% or focal to moderate immunoreactivity; iv) grade 3 corresponds to 51 to 75% or moderate to diffuse immunoreactivity; v) grade 4 corresponds to 76 to 100% or diffuse immunoreactivity [50,51,53,54].

**Statistical analyses:** we have analyzed our data using Statistical Package for Social Sciences (SPSS) software version 29. The Chi2 test was used to compare group proportions, the ANOVA test was used to compare group means, univariable logistic regression was used to evaluate the strength of association between the positive immunoreactivity to MMP-1, -2 and -9 and the appearance of POP and multivariable logistic regression was used to identify the types of MMPs most determinant in appearance of POP. The P-value < 0.05 was considered significant. The variables significantly linked to the appearance of POP in the Chi-2 and ANOVA tests have been included in the univariable analyses and those significantly linked to the POP in the univariable analyses have been included in the multivariable analyses. The adjustment was made according to the age of the patients.

**Ethical considerations:** we respected the principles of medical ethics and documentary studies rules: data were collected confidentially and treated anonymously. We obtained authorization to conduct our study from the Ethics Committee of the Kinshasa Health School and the local committee of different pilot hospitals. N°ESP/CE/19/2023 is the reference number of the approval by Ethics Committee. Each patient had freely signed an informed consent form preoperatively authorizing or permitting the use of tissues collected during surgery for research purposes.

## Results

**General characteristics of population:** the mean age of patients is 57.18±8.17 years in the study group versus 56.48±8.29 years in the comparison group and there are no statistically significant differences in the mean ages between the two groups. The mean parity of our patients is 7.76±1.04 deliveries versus 2.76±1.46 deliveries. Significant differences were noted between the mean parities in the two groups.

**Expression level of MMP (MMP-1, -2 and -9) and their association with POP:** the expression level of MMP-1, MMP-2 and MMP-9 was statistically significantly increased in the round ligaments of the study group compared with those of the comparison group for the 3 types with a mean immunoreactive of 44.10±22.04% versus 22.20±21.67% (P=0.001) for MMP-1, 39.46±24.10% versus 22.46±20.56% (P=0.001) for MMP-2 and 39.34±20.89% versus 18.38±20.54% (P=0.001) for MMP-9. In addition, statistically significant differences in positive immunoreactivity to MMP-1, MMP-2, and MMP-9 were noted in the study group compared with the comparison group (Table 1). According to unvariable analyses, statistically significant association was noted between positive immunoreactivity to MMP-1, MMP-2, and MMP-9 in the round ligaments and the occurrence of pelvic organ prolapse (Table 2). There was a significant association between positive immunoreactive to MMP-1 (AOR 5.40, 95% CI: 0.981-29.794, P:0.044) and to MMP-9 (aOR: 6.205, 95% CI: 1.467-26.239, P: 0.013) with the occurrence of POP according to multivariable analyses data in city of Kananga in the DR Congo (Table 2).

**Table 1:** immunoreactivity to matrix metalloproteinases -1, -2 and -9 in round ligaments

Types of MMPs	Settings	Study group		Comparison group		Total		P-value
MMP-1	Mean±SD	44.10±22.04		22.20±21.67		33.15±24.37		0.001
	Grade 0	2	4.00%	22	44.00%	24	24.00%	0.000
	Grade 1	14	28.00%	5	10.00%	19	19.00%	
	Garde 2	23	46.00%	20	40.00%	43	43.00%	
	Grade 3	11	22.00%	3	6.00%	14	14.00%	
	Grade 4	0	0.00%	0	0.00%	0		
	Positive immunoreactivity	48	96.00%	28	56.00%	76	76.00%	0.001
MMP-2	Mean±SD	39.46±24.10		22.46±20.56		30.96±23.85		0.001
	Grade 0	4	8.00%	20	40.00%	24	24.00%	0.003
	Grade 1	18	36.00%	10	20.00%	28	28.00%	
	Garde 2	18	36.00%	16	32.00%	34	34.00%	
	Grade3	9	18.00%	4	8.00%	13	13.00%	
	Grade 4	1	2.00%	0	0.00%	1	1.00%	
	Positive immunoreactivity	45	90.00%	28	56.00	73	73.00%	0.001
MMP-9	Mean±SD	39.34±20.89		18.38±20.54		28.86±23.15		0.001
	Grade 0	3	6.00%	26	52.00%	29	29.00	0.001
	Grade 1	17	34.00%	2	4.00%	19	19.00%	
	Garde 2	24	48.00%	18	36.00%	42	42.00%	
	Grade 3	6	12.00%	4	8.00%	10	10.00%	
	Grade 4	0	0.00%	0	0.00%	0	0.00%	
	Positive immunoreactivity	47	94.00%	24	48.00%	71	71.00%	0.001
MMP: matrix metalloproteinases; SD: Standard deviation; %: percentage; ±: more or less								

**Table 2:** risk factors associated with pelvic organ prolapse

Round ligaments						
Risk factors	Univariable analysis			Multivariable analysis		
	aOR	95% CI	P-value	aOR	95% CI	P-value
Positive immunoreactivity to MMP-1	18.85	4.21-86.28	0.001	5.40	0.981-29.794	0.044
Grade 1 and 2 of MMP-1	2.85	1.23-6.60	0.015	0.69	0.260-1.84	0.460
Positive immunoreactivity to MMP-2	8.32	2.59-2672	0.001	2.760	0.695-10.961	0.149
Grade 1 and 2 of MMP-2	2.37	1.03-5.44	0.041	0.81	0.31-2.13	0.670
Positive immunoreactivity to MMP-9	16.97	4.66-61.790.	0.001	6.205	1.467-26.239	0.013
Grade 1 and 2 of MMP-9	6.83	2.73-17.09	0.001	0.182	0.065-0.511	0.494

aOR: ajusted odd-ratio;95%; CI: 95% confidence interval; P-value: the significant p-value set to less than 0.05; POP: pelvic organ prolapse; MMP:matrix metalloproteinases

## Discussion

The objective of this study was to determine the expression rate of MMP-1, -2 and -9 in prolapsed round ligaments and to identify the types of MMPs most determining in the appearance of POP during the mass campaign in the two pilot hospitals of Saint-Georges and Bon-Berger in the city of Kananga in the DR Congo. The stromal expression levels of MMP-1, MMP-2 and MMP-9 are significantly increased in prolapsed round ligament and positive immunoreactivity to MMP-1 and -9 are more associated with POP. The expression rate of MMP-1, MMP-2 and MMP-9 is statistically significantly increased in the study group compared to the comparison group. This reflects the significant presence of increased enzymatic activity of these MMPs (MMP-1, -2 and-9) in the massive degradation of components of the extracellular matrix including collagen in patients with POP in the city of Kananga. Our results partially corroborate those of many authors including Usta *et al*. [53], Dviri *et al*. [33], and Liang *et al*. [50] who found a significant increase in the expression rate of MMP-1 in the prolapsed uterosacral and round ligaments, MMP-1 and MMP-9 in the prolapsed uterosacral ligaments and MMP-2 and MMP-9 in the prolapsed uterosacral ligaments. Strinic *et al*. [54] reported a significantly increased expression rate of MMP-1 in the uterosacral ligament of patients suffering from POP with a non-significant expression of MMP-2 while Gabriel *et al*. [36] found a significantly increased expression rate of MMP-2 with a non-significant increase in MMP-1 expression rate in the same tissues of prolapsed uterosacral ligaments. These contradictory results are contrary to ours and demonstrate the specificities of each environment and each race (black in our case).

Many authors have also studied the level of MMPs linked to prolapse in the vaginal wall of patients with POP and reported a significantly increased expression rate of MMP-2 and MMP-9 [46,55], MMP-9 but without that of MMP-2 [32,56,57] of MMP-1 and MMP-8 [39], of MMP-1, MMP-3 and MMP-9 [58], of MMP-1 and MMP-3 [59,60], of MMP-1 [34] and of MMP-10 [61] These results are partly contradictory to ours and reflect the different specificities of the organs harvested. Unlike others, Wang *et al*. in China reported significantly increased expression rate of MMP-1, MMP-2, MMP-3, and MMP-9 in the vaginal wall of patients with POP [51]. This corroborates our results despite the absence of MMP-3 assay. All these results reflect the combined action of various types of MMPs involved in the occurrence of POP in different types of pelvic organs. The positivity of MMP-1, MMP-2 and MMP-9 immunoreactivity reflects not only their synthesis, availability, but also their intense enzymatic activity in the affected tissues [30,39,50]. The positivity of MMP-1 immunoreactivity in round ligament tissue is associated with the risk of POP and significantly increases this risk by 18.85 in our city. The occurrence of POP in case of positivity of MMP-1 immunoreactivity can be explained by the fact that MMP-1 called “collagenase 1” degrades fibrillar collagen into gelatin. This leads to the decrease in the fibrillar collagen content in the connective tissues of pelvic ligaments which is the source of the reduction in the resistance to pelvic ligament traction which causes the appearance of POP [32-34,39,53,54].

The positivity of MMP-2 immunoreactivity in the round ligaments is also linked to the appearance of POP and significantly increases the risk of POP by 8 in our city. The occurrence of POP in the case of positive MMP-2 immunoreactivity is explained by the fact that MMP-2 called “gelatinase A” has two enzymatic activities, namely collagenolysis which degrades fibrillar collagen into gelatin and gelatinolysis which transforms gelatin into simple peptides recyclable by the body. This leads to a reduction in collagen content at the level of pelvic ligament with a decrease in ligament traction resistance, the source of prolapse [32,36,50,56,57]. Positive MMP-9 immunoreactivity in the round ligaments is finally associated with the occurrence of POP and multiplies the risk of POP by 17 in our town. The occurrence of POP in the case of positive MMP-9 immunoreactivity is explained by the fact that MMP-9, otherwise known as “gelatinase B”, has a collagenolytic activity allowing it to degrade fibrillar collagen into gelatin and a gelatinolytic activity at the base of the degradation of gelatin into simple peptides recyclable by the body. This results in a decrease in collagen at the level of pelvic ligament and in the resistance to ligament traction which determines the prolapse [32,36,50,56,57]. MMP-1, -2 and -9 not only degrade collagen but also other components of the extracellular matrix in particular elastin, proteoglycan at the base of the remodeling of this matrix, characteristic of pelvic organ prolapse [30,39,59,60].

Although positive immunoreactivities to MMP-1, MMP-2 and MMP-9 are significantly associated with the occurrence of prolapse, the positivity of MMP-1 and MMP-9 is the most determining in the occurrence of POP in the city of Kananga. MMP-1, MMP-2 and MMP-9 are cross-activated by MMP-3, MMP-7, MMP-10, interleukin-1, TNF-α whose expression is also increased in patients with POP [30,39,46,51,62] and are inhibited by tissue inhibitors of MMPs (TIMPs), estrogens, progestins whose expression level is decreased in POP [30,35,39,47,50]. Many epidemiological factors associated with pelvic organ prolapse, including obstetric trauma, stimulate these MMPs by acting through an activating factor (interleukin-1) or by suppressing inhibitory factors such as menopause [35,47,50]. Our results serve as a basis on the one hand for studies to search for factors associated with increased expression rate of MMP-1, -2 and -9 in patients with POP and on the other hand for experimental research studies for inhibitory evidence of estrogen and progesterone on MMP-1, MMP-2 and MMP-9 in our city (given that this inhibitory evidence has been provided by studies conducted elsewhere) with a view to adding these hormones in the prevention of POP in women at risk in our city of Kananga as is practiced elsewhere [30,35,39,42,47,50]. The weaknesses of this study are that it did not study the expression rate of MMP-1, -2 and -9 genes in women with POP, and did not study other MMPs associated with prolapse such as MMP-8, MMP-3, MMP-7 and MMP-10. The strengths of our study are that it is the first to determine the expression rate of MMP-1, -2 and -9 in hospital settings in the city of Kananga, the DR Congo and Africa; and that it is the basis for further studies to investigate the inhibitory role of estrogen-progestins and factors associated with high levels of MMP-1, -2 and -9 in the city of Kananga.

## Conclusion

Tissue levels of MMP-1, -2 and -9 are significantly increased in the round ligament tissue of patients with POP. The positivity of immunoreactivity is significantly associated with POP for the three MMPs studied but more determinant by MMP-1 and MMP-9 in our city. Our results serve as a basis, on the one hand, for studies of research of factors associated with the increase in the expression rate of MMP-1, -2 and -9 in patients with POP and on the other hand, for experimental research studies of inhibitory evidence of estrogens and progesterone on MMP-1, -2 and -9 in our city with a view to adding these hormones in the prevention of POP in women at risk in city of Kananga in DR Congo.

### What is known about this topic

- Pelvic organ prolapse is a pathology of ligamentous connective tissues;
- Factors associated with pelvic organs prolapse in the city of Kananga are intense physical work, multiparity, malnutrition (BMI less than 18.5 kg/m^2^), vaginal delivery, obstetric trauma and menopause and fetal macrosomia;
- Lack of data on the expression of matrix metalloproteinases linked to pelvic organ prolapse in our city of Kananga.

### What this study adds

- The expression level of matrix metalloproteinases -1, -2 and -9 is significantly increased in patients with pelvic organ prolapse;
- Immunoreactivities to matrix metalloproteinases -1, -2 and -9 are significantly linked to pelvic organ prolapse but those to matrix metalloproteinases -1 and -9 are more decisive;
- These results will serve as the basis for experimental research studies of inhibitory evidence of estrogens and progesterone on matrix metalloproteinases -1, matrix metalloproteinases -2 and matrix metalloproteinases -9 linked to pelvic organs prolapse in order to contribute to improving its management in our environment.
